# Functional ferroelectric tunnel junctions on silicon

**DOI:** 10.1038/srep12576

**Published:** 2015-07-28

**Authors:** Rui Guo, Zhe Wang, Shengwei Zeng, Kun Han, Lisen Huang, Darrell G. Schlom, T. Venkatesan, A Ariando, Jingsheng Chen

**Affiliations:** 1 Department of Materials Science and Engineering, National University of Singapore, 117575, Singapore; 2 NUSNNI-Nanocore, National University of Singapore, 117411, Singapore; 3 Department of Materials Science and Engineering, Cornell University, Ithaca, NY 14853, USA; 4 Department of Physics, National University of Singapore, 117542, Singapore; 5 Kavli Institute at Cornell for Nanoscale Science, Ithaca, NY 14853, USA; 6 Department of Electrical and Computer Science Engineering, National University of Singapore, 117576, Singapore; 7 School for Integrative Sciences and Engineering, National University of Singapore, 117456, Singapore

## Abstract

The quest for solid state non-volatility memory devices on silicon with high storage density, high speed, low power consumption has attracted intense research on new materials and novel device architectures. Although flash memory dominates in the non-volatile memory market currently, it has drawbacks, such as low operation speed, and limited cycle endurance, which prevents it from becoming the “universal memory”. In this report, we demonstrate ferroelectric tunnel junctions (Pt/BaTiO_3_/La_0.67_Sr_0.33_MnO_3_) epitaxially grown on silicon substrates. X-ray diffraction spectra and high resolution transmission electron microscope images prove the high epitaxial quality of the single crystal perovskite films grown on silicon. Furthermore, the write speed, data retention and fatigue properties of the device compare favorably with flash memories. The results prove that the silicon-based ferroelectric tunnel junction is a very promising candidate for application in future non-volatile memories.

Over the past two decades, information technologies (ITs) have evolved from the PC era, to mobile era and now to the consumer-mobile era. Different eras have emphasized different requirements for memories, which stimulated research along the directions of non-volatile, high density, high speed and low power consumption. Especially, energy efficient non-volatile memory based on voltage (electric field) induced/assisted writing has recently received intensive attention^1^,^2^. Ferroelectric random-access memories (FeRAM) have characteristics such as non-volatility, high speed and low writing energy since the spontaneous polarization is switched by an external electric field^3^,^4^. However, the capacitive readout of the information is destructive and a re-write process is required after the readout. Recent experimental and theoretical studies of ferroelectric oxide thin films showed that ferroelectricity can persist down to a few unit cells^5^,^6^, which opened a new paradigm for non-destructive FeRAM which utilizes an ultrathin ferroelectric layer as the tunnel barrier in metal/ferroelectric/metal junctions. The asymmetry of screening length of two electrodes leads to the change of the electric resistance when the ferroelectric polarization is switched. This device concept is called ferroelectric tunnel junction (FTJ), and the phenomenon that the electric resistance is modulated by the ferroelectric polarization switching is called tunnelling electroresistance (TER) effect^7^,^8^,^9^. Very large TER effects have been reported in FTJs with BaTiO_3_ (BTO)^10^,^11^,^12^,^13^,^14^,^15^,^16^ and Pb_1-x_Zr_x_TiO_3_^17^,^18^,^19^ ferroelectric tunnel barrier. Writing time as fast as 10 ns and writing energy smaller than 10 fJ/bit were achieved in FTJs^14^. However, those FTJ devices were demonstrated on perovskite heterostructures with cubic SrTiO_3_ (STO) (001)^11^,^15^,^17^,^18^,^19^ or orthorhombic NdGaO_3_ (110)^10^,^12^,^13^,^14^ single crystal substrates, which provide the compressive strain to BTO/PZT layer to maintain the ferroelectricity of the ultra-thin ferroelectric tunnel barrier. The usage of perovskite single crystal substrates is incompatible with the current memory fabrication process which is based on silicon substrates. The capability of fabricating FTJs on silicon substrates therefore becomes essential for the practical application of FTJs as memories. Recently, the non-volatile memory function of FTJs on silicon substrate was demonstrated by using conductive atomic force microscopy (c-AFM)^20^. However, a real FTJ device demonstration as well as the performances of FTJ devices on silicon substrate such as the writing speed, endurance and fatigue are still lacking and warrant investigation.

Motivated by practical application, in this work, we demonstrate epitaxial FTJ devices on Si substrates with BTO tunnel barrier with thickness varying from 1.5 to 4 nm. The dependence of TER on BTO thickness, the writing time, the endurance and fatigue of the FTJs device were investigated. The structure of the device is Pt/BTO/La_0.67_Sr_0.33_MnO_3_ (LSMO)/STO/Si, as shown in Fig. 1 (a). An epitaxial layer of STO was deposited on Si (001) substrate as a template (see Methods for details). We use LSMO as the bottom electrode. After the deposition of LSMO, an ultrathin layer of BTO was grown as the ferroelectric tunnelling barrier layer. Following the BTO deposition, an array of 5 μm × 5 μm Pt electrodes (25 nm thick) was patterned on top for subsequent electrical characterizations.

## Results

### Microstructure Analysis of the Tunnel Junctions

**Figure 1:**
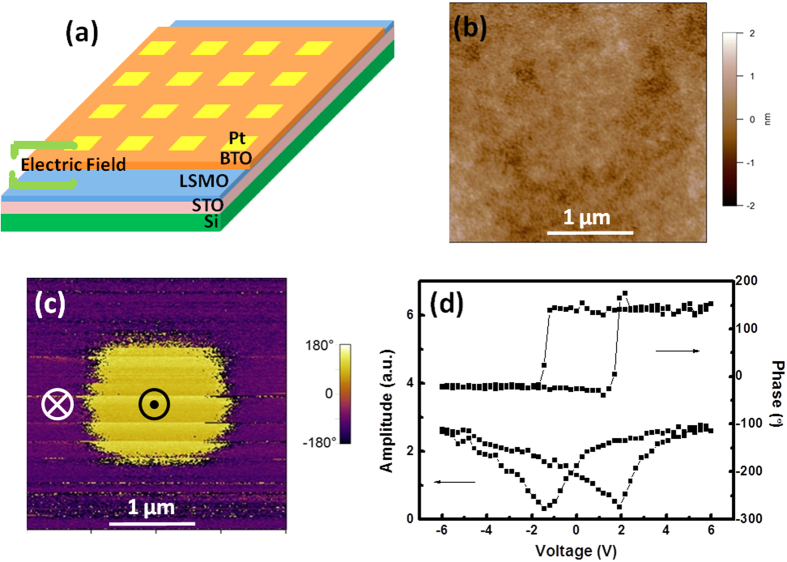
(**a**) Schematic drawing of the tunnel junction structure. (**b**) Topography of the BTO thin film. (**c**) Out-of-plane PFM phase image of BTO thin film. A 2-μm × 2-μm square region with opposite polarization direction was written by scanning the area with a DC bias (−4 V) applied to the AFM tip. (**d**) Local PFM hysteresis loops of BTO thin film.

Piezoelectric force microscopy (PFM) was performed to characterize the ferroelectric domain structure of BTO films in a separate FTJ device without Pt electrode. Figure 1 (b) show the topography, and out-of-plane (OOP) PFM phase images of BTO film with the thickness of 2 nm grown on LSMO/STO/Si heterostructure. The OOP PFM phase image showed uniform contrast; while there was no signal in in-plane PFM images (thus were not shown here). In our PFM system, the yellow contrast in OOP image means the polarization points up (upward), while the purple contrast means the polarization points down (downward). So the PFM result indicates a single downward domain structure in BTO films. To switch the downward polarization, a negative DC bias of −4 V was applied to a selected area through the tip. After the switching, the downward domain changed into upward domain, which reveals a 180° switching path as shown in Fig. 1 (c). Figure 1 (d) shows the PFM hysteresis loops of the 2 nm thick BTO film, indicating its ferroelectric nature. According to the basic quantum mechanism, the tunneling transmittance depends exponentially on the barrier width as well as the barrier height. It has already been reported that a giant TER was achieved by modulating the barrier width using n-type doped Nb:SrTiO_3_ as bottom electrode through ferroelectric field effect^16^. In this work, hole doped LSMO is used as the bottom electrode. Therefore, in our case, ON state of the tunnel junction is achieved by a narrower barrier width when the polarization of BTO points up (hole accumulation state at LSMO interface), while OFF state is realized by a wider barrier width when the polarization points downward (hole depletion state at LSMO interface).

**Figure 2:**
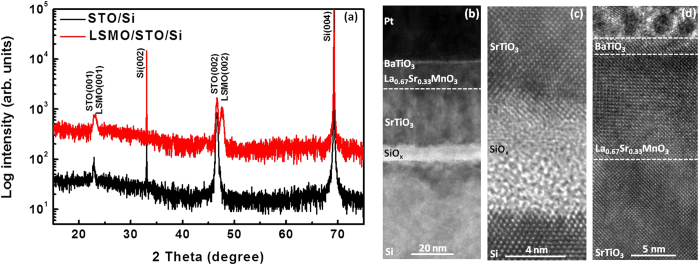
(**a**) XRD patterns of STO/Si and LSMO/STO/Si structures. (**b**) HRTEM image of the whole structure of the device. The thin write layer between Pt and LSMO is BTO thin film, and a layer of amorphous SiO_x_ is between Si and crystalline STO. (**c**) HRTEM image of STO/SiO_x_/Si. The interfaces between the amorphous SiO_x_ layer and crystalline Si and STO layers are clearly shown. (**d**) HRTEM image of Pt/BTO/LSMO/STO. The dashed lines show the interface of PT/BTO, BTO/LSMO, and LSMO/STO, respectively.

The high-resolution X-ray diffraction (XRD) spectra of the heterostructures (STO/Si and LSMO/STO/Si) are shown in Fig. 2 (a). The calculated average c lattice constant of STO is about 0.4 nm. The elongated c lattice is due to the large compressive strain of about 1.7% between STO and Si with the STO unit cell rotated by 45° relative to the Si conventional unit cell. The full width at half maximum (FWHM) of the rocking curve of STO/Si XRD (002) peak (Supplementary figure S1) is about 0.37°. These XRD spectra suggest a good epitaxial growth of the perovskite film on Si. Figure 2 (b–d) shows the schematic drawing of the cross-sectional structure and the cross-sectional high resolution transmission electron microscope (HRTEM) images of the tunneling device. The thickness of BTO tunnel barrier, LSMO bottom electrode, and STO template layer is about 2, 12.3, and 28 nm, respectively. Between the crystalline Si substrate and the crystalline STO layer, there is an amorphous layer of SiO_x_ with the thickness around 8–9 nm. This amorphous layer forms during the oxide films deposition process^21^,^22^. To be specific, there are two processes during the growth that may lead to the formation of the amorphous layer. Firstly, during the growth of STO template layer, the oxygen might diffuse into the STO/Si interface and oxidize the Si substrate as the STO layer grew thicker, although the STO layer preserves the original epitaxial registry during the subsequent growth. Secondly, the following LSMO and BTO layers were deposited at 650 °C with an oxygen pressure of 300 mTorr and 5 mTorr, respectively. Under these growth conditions, the oxygen could diffuse through the STO layer and further oxidize the Si substrate. The interfaces between amorphous SrO_x_ layers and crystalline Si and STO layers are shown in Fig. 2 (c). The transitions between crystalline and amorphous phases can be clearly observed. Figure 2 (d) shows the interfaces of LSMO/STO, and BTO/LSMO heterostructures. These interfaces are almost atomically sharp, which proves the high epitaxial quality of the perovskite films. The Fast Fourier Transformation (FFT) patterns of BTO, LSMO, STO, and Si TEM images are shown in Supplementary figure S2. The calculated average lattice parameter ratio *c/a* of STO layer is about 1.02. The elongated *c* lattice is due to the compressive strain between the film and Si substrate, which is consistent with the XRD result. The in-plane lattice constant *a* of LSMO is around 0.383 nm, which will cause a compressive strain to BTO (the in-plane lattice constant of bulk BTO is 0.399 nm) layer due to the lattice mismatch. The calculated tetragonality (*c/a* ratio) of BTO is about 1.07, which is much larger than the bulk value of 1.01. The increased tetragonality can greatly enhance the polarization of BTO thin film and favors the maintenance of ferroelectricity for an ultra-thin BTO layer^23^.

### Basic Properties of the Tunnel Junctions

**Figure 3:**
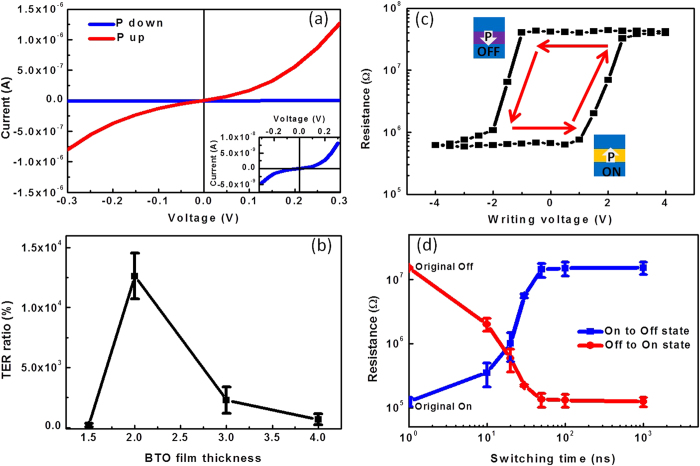
(**a**) *I*–*V* curves of the tunnel junction (2 nm BTO) with different BTO polarizations. The inset shows the *I-V* curve of the OFF state (P down). (**b**) *R*-*V* switching curve of the junction (2 nm BTO). (**c**) TER ratios of the tunnel junctions as the function of BTO thickness. (**d**) Electrical resistance as functions of switching time for both upward and downward polarization states of the junctions (switching pulse ±3 V). The two points on the y-axis which correspond to 10^0^ of x-axis represent the original OFF and ON states, respectively.

The current-voltage (*I*–*V*) curves for both upward and downward polarization states of the tunneling device with 2 nm BTO are shown in Fig. 3 (a). The applied voltage is termed as positive (negative) if a positive (negative) bias is applied to the top Pt electrode. After poling the polarization up (down) by applying a voltage pulse of −3 V (+3 V), the tunneling currents are measured at room temperature within low dc range, from −0.3 V to 0.3 V, which is certainly lower than the coercive voltage (*V_C_*). For both on and off states, the *I*–*V* curves are nonlinear, which is an indication of elastic electron tunneling. With a reading voltage of 0.3 V, the currents for the ON and OFF states of this device with 2 nm BTO layer reach 1.28E-6 A and 8.03E-9 A, respectively, giving a TER ratio of 15800%. To reveal the influence of the ferroelectric thin film thickness on the tunneling effect, the dependence of the TER ratio on the BTO film thickness was studied, and the results are shown in Fig. 3 (b). The largest TER ratio was achieved when the BTO thickness is 2 nm. In this work, five devices were measured, the average TER ratio being around 12000%. When the BTO thickness decreases to 1.5 nm, the TER ratio drops rapidly and almost disappears. This may be due to the fact that the ferroelectricity of BTO film becomes very weak or even disappears when its thickness approaches 1.5 nm. This result is consistent with the report that the critical thickness for ferroelectricity of BTO film on STO substrate is around 1.6 nm^24^. However, we believe this may also be due to the increased leakage probability for thinner BTO layers where by reducing the device size significant TER was seen even with BTO layer thicknesses of 0.8 nm (2 unit cells)^25^. When the thickness of BTO goes up to 3 and 4 nm, the TER ratio also decreases drastically. This may be because with the increased barrier width, the tunneling effect would no long dominate and other mechanisms start to play a role. In the following discussions, we focus on the FTJ device with BTO thickness of 2 nm.

The non-volatile electrical resistance switching (*R*-*V* loop) of Pt/BTO/LSMO/STO/Si device with BTO thickness of 2 nm is illustrated in Fig. 3 (c). Similar to the PFM hysteresis loop of ferroelectric in Fig. 1 (d), a clear hysteretic variation of the tunneling resistance is observed. This proves that it is the polarization that causes the change of the resistance. The coercive voltage is below 2 V. It is shown that with the positive and negative voltage pulse switching the device shows the high and low resistance states, respectively. A positive pulse above coercive voltage switches the polarization of BTO to downward direction towards the bottom electrode and vice versa. The high (low) resistance at the positive (negative) voltage can be explained by the change of tunnel barrier width through the depletion (accumulation) of the hole concentration in LSMO bottom electrodes.

### Writing Speed, Data Retention, and Fatigue Performance of the Tunnel Junctions

To evaluate the writing speed of the ferroelectric tunnel junction on silicon substrate, the dependence of the switching pulse width on the electrical resistance of the FTJ device were measured and the results are shown in Fig. 3(d). Square pulses with the width from 10 ns (the limit of our pulse generator) to 1 μs are used to control the polarization direction of the BTO tunnel barrier, and the *I*–*V* curves were measured subsequently to detect the electrical resistance of the device (Supplementary figure S3). After applying ±3 V pulses, the spontaneous polarization starts to switch at 10 ns. At 50 ns, the polarization is fully reversed and the ON and OFF electrical resistances saturate at the expected values. The results demonstrate that the ferroelectric tunnel junctions can be written within 50 ns. However, this is by no means the limit. Shorter writing time is expected if higher pulse voltage is used^26^. In fact, it has already been reported that polarization switching of ferroelectric Pb(Nb_0.04_Zr_0.28_Ti_0.68_)O_3_ thin film can be realized with a voltage pulse less than 1 ns generated by a semiconductor photoconductive switch^27^. To read the ON and OFF states of the tunnel junctions, we can detect the electrical resistance by simply measuring the *I*–*V* curves. Therefore the reading process is non-destructive since it does not require the switching of the ferroelectric polarization.

**Figure 4:**
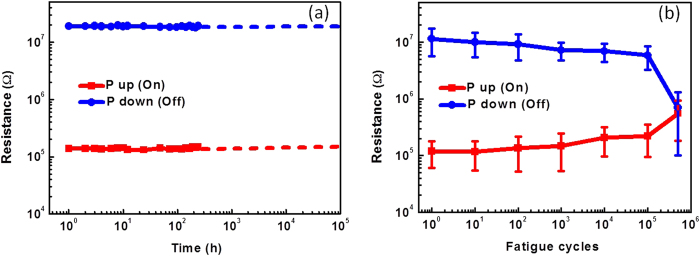
(**a**) Resistance for both polarization directions show negligible change after 10 days. (**b**) Resistances measured after repetitive switching by pulses of ±3 V, 0.05 ms reveal no fatigue after 10^5^ cycles.

Long data retention and superior fatigue performance are two critical requirements for the application of non-volatile memory devices. We have monitored the resistance of several tunnel junctions for one week, and no deterioration in the signal has been observed (Fig. 4 (a)). The junctions were under open boundary condition during the periods between the read-out measurements. By extrapolation, we can estimate that the TER ratio of the junctions could be maintained over 10000% for more than 10 years. Furthermore, the tunnel junctions have been subjected to bipolar switching for up to 10^5^ cycles, as shown in Fig. 4 (b). The fatigue properties were measured by applying bipolar electrical pulses of ±3 V (pulse width of 0.05 ms) with different durations to the devices. After each cycling, the devices were set to ON and OFF states again, and *I*–*V* curves were measured accordingly to test the corresponding resistance state (Supplementary figure S4). As shown in Fig. 4 (b), after 10^5^ cycles, the tunnel junctions can still be switched to the original ON and OFF states, with only a slightly decreased TER ratio. This proves that the polarization of BTO thin film is still switchable after 10^5^ cycles. With further electrical cycling, the tunnel junctions could not function any more, which might be due to domain wall pinning of the BTO thin film. It is well known that oxide electrodes can mitigate the fatigue problem in ferroelectric oxide, because they can reduce the oxygen vacancy concentration at the film/electrode interface, leading to less domain pinning^28^. This suggests that fatigue properties of tunnel junctions might be improved further by fine tuning the interfaces between the electrodes and tunnel barrier. The endurance of current FTJ device in this work is larger than that of the NAND flash memory (around 10^4^ cycles).

## Discussion

In summary, we report here a functional non-volatile FTJ grown directly on Si substrate, with a TER ratio over 10000%. The fast writing speed (within 50 ns by ±3 voltage pulses), long data retention and good fatigue performance of the tunneling devices compare favorably with flash memories which dominate in the current non-volatile memory market. All our results show that ferroelectric tunnel junction is a very promising candidate for future silicon-based non-volatile resistive memories with high speed and low power consumption.

## Methods

### Device preparation

STO template layer was fabricated using a two-step growth method by molecular beam epitaxy (MBE) technique. Before the growth, SiO_2_ layer on top of the Si substrate was deoxidized. This was achieved by using a Sr-assisted deoxidization process^29^. After heating the Si substrate to 600 °C, 2 monolayers of metal Sr was deposited. Afterwards, Si was heated up to 800 °C with ~10 min wait until a clear RHEED pattern of crystalline Si (100) surface was seen. After cooling the Si substrate to 600 °C, more Sr was deposited until a clear 2x reconstruction pattern was observed from RHEED. Following the deoxidization process, 2.5 u.c. of STO was grown at 350 °C with the oxygen partial pressure in the mid to upper 10E-8 Torr range. The as-grown 2.5 u.c. of STO was annealed at 600 °C in vacuum for ~10 min for recrystallization. This process was repeated three times until we got a 7.5 u.c. STO film on Si (1st step)^30^. With the 7.5 u.c. of epitaxially grown STO, another 25 nm STO was grown at ~550 °C using the same oxygen partial pressure without annealing (2nd step).

The following LSMO and BTO layer were deposited by pulsed laser deposition (PLD) technique. LSMO was deposited at a substrate temperature of 600 °C with an oxygen partial pressure of 300 mTorr, and BTO films were deposited subsequently at 650 °C with an oxygen partial pressure of 5 mTorr. The laser energy density was fixed at 1 J/cm^2^ for all depositions with a frequency of 3 Hz. After the deposition, the films were cooled down to room temperature at an oxygen pressure of 100 Torr. The cooling rate was 5 °C/min till 300 °C, and then 15 °C/min to room temperature.

After the deposition of BTO, 5 μm × 5 μm top electrodes were prepared by photolithography. After the photolithography, Pt electrodes were deposited by PLD at room temperature. The remaining photoresist was stripped by a remover solution (MicroChem).

### Electrical characterizations

Piezoelectric force microscopy (PFM) measurement was carried out on a commercial atomic force microscope (Asylum Research MFP-3D) using Pt/Ti-coated tip. The measurement was performed under contact mode with an AC voltage applied to the probe tip. The scan rate was kept at 0.5 μm/s. *I*–*V* curves were measured using a metre/direct current (DC) voltage source (Keithley 2635B) on a low noise probe station. Fatigue properties and switching experiments were carried out using a commercial ferroelectric tester (Radiant Technologies, Inc.) and a pulse generator (Genetron PSPL Model 10, 700 A pulse generator).

## Additional Information

**How to cite this article**: Guo, R. *et al.* Functional ferroelectric tunnel junctions on silicon. *Sci. Rep.* **5**, 12576; doi: 10.1038/srep12576 (2015).

## Supplementary Material

Supplementary Information
