# Supplementary material for: Functional ferroelectric tunnel junctions on silicon
Source: Sci Rep. 2015 Jul 28;5:12576. doi: 10.1038/srep12576 (PMC4517170; doi:10.1038/srep12576)
Supplement: Supplementary Information [file srep12576-s1.doc]

Functional ferroelectric tunneling junctions on silicon

Supplementary information

Rui Guo^1,2^, Zhe Wang^3^, Shengwei Zeng^2,4^, Kun Han^2,4^, Lisen Huang^1^, Darrell G. Schlom^3^, T.Venkatesan^1,2,4,5*^, Ariando^2,4*^, Jingsheng Chen^1*^

^1^Department of Materials Science and Engineering, National University of Singapore, 117574, Singapore. ^2^NUSNNI-Nanocore, National University of Singapore, 117411 Singapore.

^3^Department of Materials Science and Engineering, Cornell University, Ithaca, NY 14853, USA.

^4^Department of Physics, National University of Singapore, 117542 Singapore.

^5^Department of Electrical and Computer Engineering, National University of Singapore, 117576 Singapore


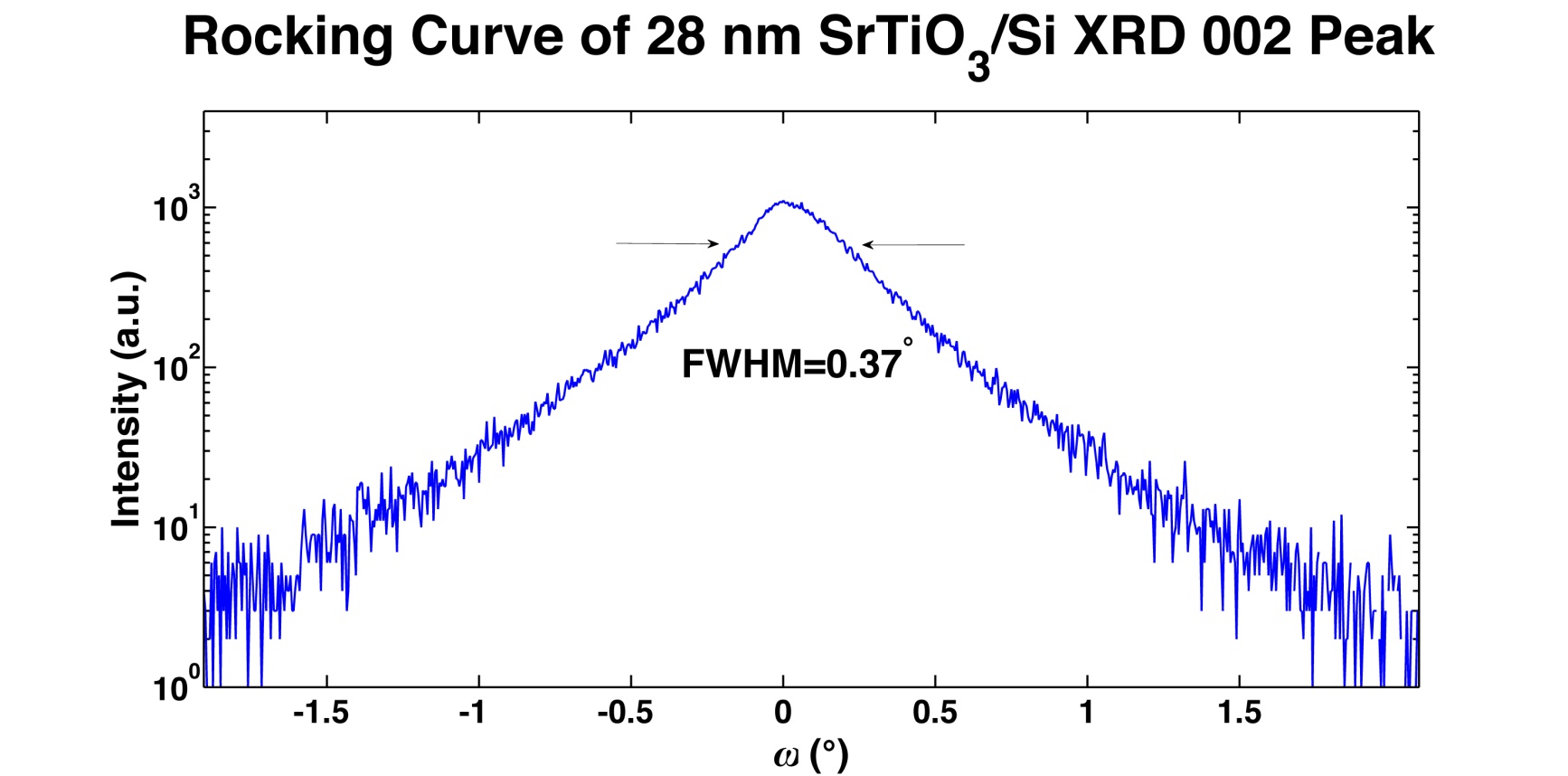


Figure 1. Rocking curve in ω of the out-of-plane SrTiO_3_/Si XRD (002) peak. The full width at half maximum (FWHM) is about 0.37°.


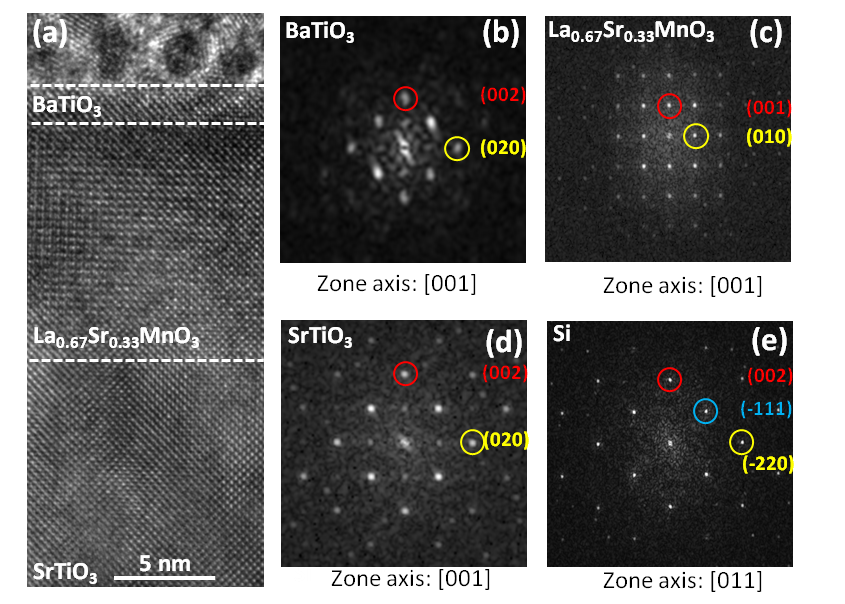


Figure 2. TEM Fourier diffraction patterns of (b) BTO, (c) LSMO, (d) STO thin films, and (e) Si substrate. (b)-(d) are obtained from the TEM image (a), and (e) is taken from the Si substrate. Lattice parameters of the three layer thin films are calculated from the diffraction patterns. Lattice c and a are 0.398 and 0.390 nm for STO, 0.395 and 0.383 nm for LSMO, and 0.416 and 0.388 nm for BTO, respectively. So the tetragonality (c/a ratio) of BTO is about 1.07.


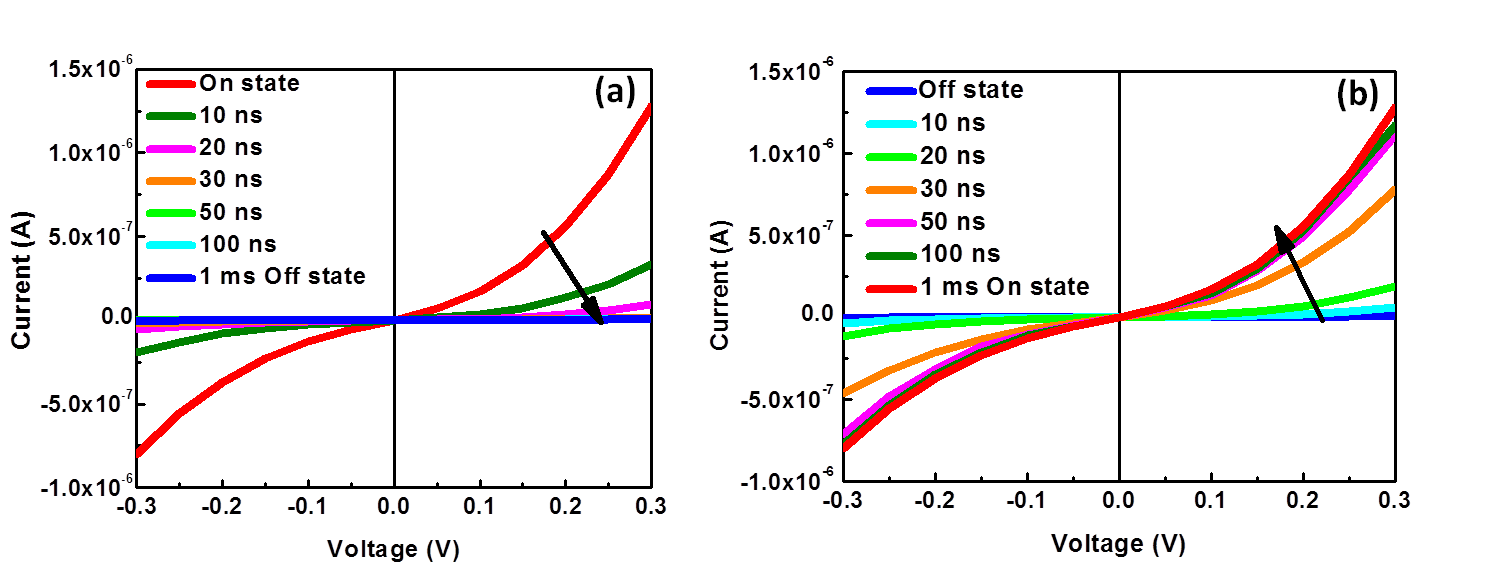


Figure 3. (a) *I*-*V* curves of one sample as functions of the switching time from On state to Off state, and (b) *I*-*V* curves of the same sample as functions of the switching time from Off state to On state. The switching pulse is ± 3 V, respectively. After 50 ns, the *I*-*V* curves almost keep the same with the increase of the switching time, which suggests the fully switching of the polarization.


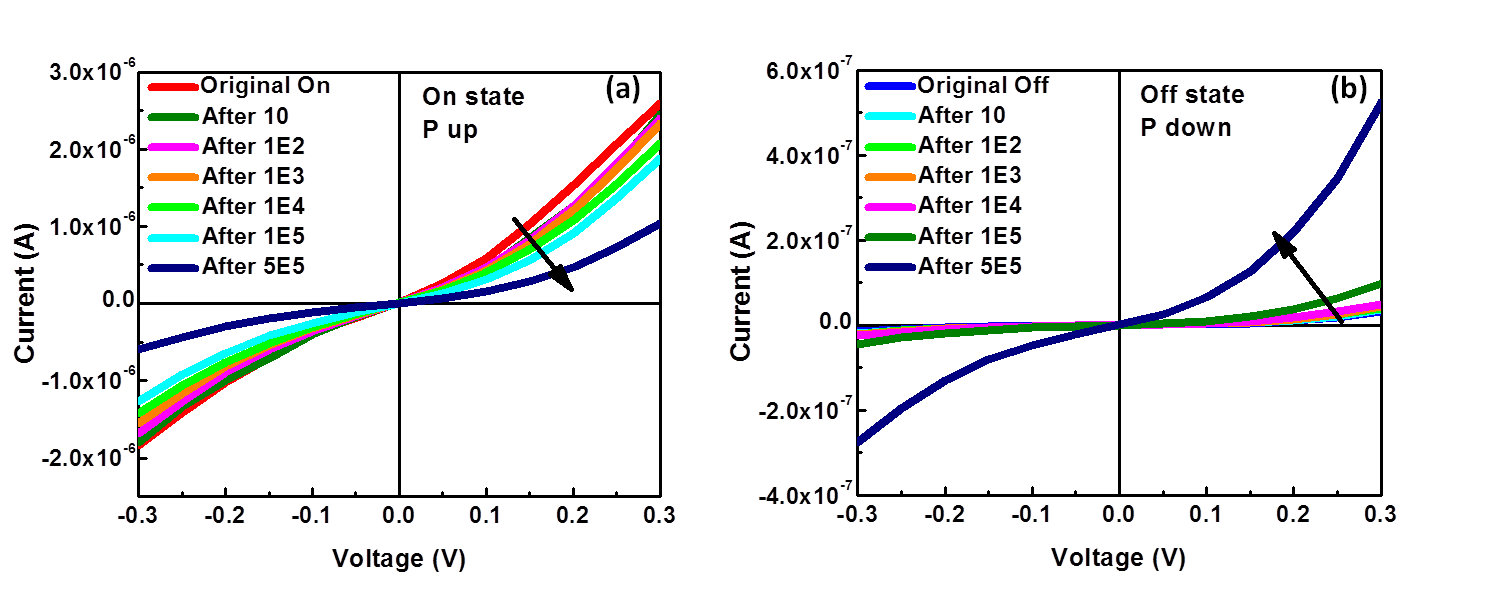


Figure 4. *I*-*V* curves measured at different fatigue cycles by pulses of ±3 V, 0.05 ms, of (a) one device with upward polarization, and (b) one device with downward polarization. Up to the cycles of 1E5, the polarization of the devices can still be switchable, giving a slightly changed TER ratio. After the cycles of 5E5, fatigue happens, and *I*-*V* curves of the two states do not differ much any longer. Device can sustain the switching cycles of 10^5^.
